# Diabetic Nephropathy Amelioration by a Low-Dose Sitagliptin in an Animal Model of Type 2 Diabetes (Zucker Diabetic Fatty Rat)

**DOI:** 10.1155/2011/162092

**Published:** 2011-11-30

**Authors:** Cristina Mega, Edite Teixeira de Lemos, Helena Vala, Rosa Fernandes, Jorge Oliveira, Filipa Mascarenhas-Melo, Frederico Teixeira, Flávio Reis

**Affiliations:** ^1^Laboratory of Pharmacology & Experimental Therapeutics, Institute for Biomedical Research on Light and Image (IBILI), Medicine Faculty, Coimbra University, 3000-548 Coimbra, Portugal; ^2^Agrarian School of Viseu, Polytechnic Institute of Viseu, 3500-606 Viseu, Portugal; ^3^Educational, Technologies and Health Study Center, Polytechnic Institute of Viseu, 3504-510 Viseu, Portugal

## Abstract

This study was performed to assess the effect of chronic low-dose sitagliptin, a dipeptidyl peptidase 4 inhibitor, on metabolic profile and on renal lesions aggravation in a rat model of type-2 diabetic nephropathy, the Zucker diabetic fatty (ZDF) rat. Diabetic and obese ZDF (fa/fa) rats and their controls ZDF (+/+) were treated for 6 weeks with vehicle (control) or sitagliptin (10 mg/kg/bw). Blood/serum glucose, HbA1c, insulin, Total-c, TGs, urea, and creatinine were assessed, as well as kidney glomerular and tubulointerstitial lesions (interstitial fibrosis/tubular atrophy), using a semiquantitative rating from 0 (absent/normal) to 3 (severe and extensive damage). Vascular lesions were scored from 0–2. Sitagliptin in the diabetic rats promoted an amelioration of glycemia, HbA1c, Total-c, and TGs, accompanied by a partial prevention of insulinopenia. Furthermore, together with urea increment prevention, renal lesions were ameliorated in the diabetic rats, including glomerular, tubulointerstitial, and vascular lesions, accompanied by reduced lipid peroxidation. In conclusion, chronic low-dose sitagliptin treatment was able to ameliorate diabetic nephropathy, which might represent a key step forward in the management of T2DM and this serious complication.

## 1. Introduction

Type 2 diabetes mellitus (T2DM) is an increasing health problem, with increasing prevalence and incidence, according all the estimates worldwide [1]. The core pathophysiology of type 2 diabetes (T2DM) has been attributed to the classic triad of decreased insulin secretion, increased insulin resistance, and elevated hepatic glucose production. Further mechanisms have also key relevance, including those related with the fat cell (accelerated lipolysis), the gastrointestinal tract (incretin deficiency/resistance), the pancreatic *α*-cell (hyperglucagonemia), the kidney (increased glucose reabsorption), as well as the brain (insulin resistance), now referred to as the “ominous octet” [2]. The main problem of T2DM management is its serious micro- and macrovascular complications, which include, among others, diabetic nephropathy [3]. The incidence of T2DM is rapidly increasing, as is the prevalence of cardiovascular disease (CVD) and chronic kidney disease (CKD) resulting from diabetic complications [4, 5]. Diabetes remains the single most important cause of kidney failure, and diabetic nephropathy is a major microvascular complication of diabetes and progression to end-stage renal disease (ESRD) in different regions of the world [6, 7], accounting for approximately one-third of all cases of end-stage renal disease.

There is emerging evidence that microvascular disease begins prior to the onset of diabetes, and this occurs with microalbuminuria and decreased renal function. Experimental and clinical studies showed an adaptive response by the kidney to conserve glucose, which is essential to meet the energy demands of the body [8–11]. In the diabetic patient, instead of dumping glucose in the urine to correct hyperglycaemia, the kidney chooses to hold on to glucose. Even worse, the ability of the diabetic kidney to reabsorb glucose appears to be augmented by an absolute increase in the renal reabsorptive capacity for glucose [12, 13]. The hyperglycaemic profile is aggravated by oxidative stress damage and inflammation, as well as by overactivity of the renin-angiotensin-aldosterone system (RAAS) and alteration of the extracellular matrix protein synthesis by glomerular epithelial cells, which contributes to further aggravate diabetic nephropathy [14–17].

Evidence is available that long-term maintenance of normal or near-normal glucose levels using pharmacological means is protective in diabetic patients, improving microvascular disease and reducing both morbidity and mortality [18–20]. Traditionally, noninsulin-dependent T2DM is pharmacologically managed with oral antidiabetic agents from several different classes, which includes agents that increase insulin secretion, improve insulin action, and delay absorption of carbohydrates. The more recent incretin-based therapies address a previously unmet need in the diabetic therapeutic approach by modulating glucose supply [21–23]. Their pharmacological action is based on gut incretin hormones, the glucose-dependent insulinotropic peptide (GIP), and the glucagon like peptide-1 (GLP-1), which appear to be malfunctioning in T2DM and have important effects on insulin and glucagon secretion [24, 25]. Sitagliptin is one of the best known incretin enhancers (or gliptin), which increase incretin contents due to the inhibition of dipeptidyl peptidase-4 (DPP-4) activity, which is responsible for the degradation of GLP-1 [23, 26–28]. Even though there is a patent association in observational studies between hyperglycaemia and diabetic complications, the benefits of a strict glycaemic control on micro- and macrovascular complications have been questioned. Therefore, the benefits of glucose reduction seem to be, at least partly, minimized by the side effects of the glucose-lowering antidiabetic agents, including hypoglycaemia, weight gain, and fluid retention. In this context, new therapeutic options with fewer side effects are advisory, and the appearance of incretin-based therapies is a hope. However, clinical studies with renal end points using these agents are lacking as well as animal studies assessing the influence of these drugs on renal function and lesion.

Rodent models of T2DM are frequently used to clarify the mechanisms responsible for the pathophysiology of diabetes evolution, as well as its complications. The Zucker diabetic fatty (ZDF) rat has a mutation in the gene coding the leptin receptor (fa/fa) that results in obesity, insulin resistance, reduced glucose tolerance, hypertension, and renal and cardiovascular (CV) disease, thus developing a phenotype very similar to humans with T2DM, including the existence of diabetes nephropathy [29–31]. Our group has previously reported that a chronic low-dose sitagliptin treatment promotes not only a reduction of hyperglycaemia, but also other protective actions (including antioxidant and anti-inflammatory actions) [32]. Considering the extra-pancreatic effects of incretins, namely, the GLP-1 ability to positively modulate the function of other tissues [33, 34], it seems important to evaluate the effects of sitagliptin in diabetic nephropathy as well as to characterize the nature of the putative benefit. 

Concerning the management of diabetic nephropathy, the ability of antidiabetic drugs to ameliorate renal microvascular disease might be as important as their capability to control glucose. While the lowering glucose effects of incretin-dipeptidyl peptidase-IV (DPP-4) are well known, the effects on the kidney remain to be elucidated. Thus, the present study aimed to evaluate whether sitagliptin can prevent the development of renal dysfunction in diabetic ZDF rats.

## 2. Materials and Methods

### 2.1. Animals and Experimental Design

Male ZDF rats (ZDF/Gmi, fa/fa) and their littermates (ZDF/Gmi, +/+) were purchased from Charles River Laboratories (Barcelona, Spain) at 6 weeks of age. Rats were properly housed, handled daily, and kept in a controlled standard temperature (22-23°C), humidity (60%), and light-dark cycles (12/12 h). Throughout the experiment, the animals were provided with distilled water *ad libitum* and rodent maintenance chow (A-04 Panlab, Barcelona, Spain, containing 15.4% of protein and 2.9% of lipids). The chow was adapted to the animal's body weight (BW): 100 mg/g. Animal experiments were conducted according the European Council Directives on Animal Care and the National Laws. 

When aged 20 weeks, eight lean control ZDF/Gmi (+/+) and eight obese diabetic ZDF (fa/fa) rats were sacrificed for tissue collection. The remainder diabetic ZDF (fa/fa) rats were divided the following two subgroups (*n* = 8 rats each) for a treatment period between 20 (T0) and 26 (Tf) weeks of age: a control and a treatment group, receiving, respectively, by oral gavage, once a day (6:00 PM), for 6 weeks, the vehicle (orange juice) and sitagliptin (10 mg/kg/BW). The same procedures were adopted with the lean nondiabetic ZDF (+/+) control rats. The ZDF (+/+) control group under sitagliptin treatment showed no relevant differences when compared with the ZDF (+/+) control rats under vehicle, and thus, the results were excluded from tables and figures in order to facilitate data comparison and interpretation. Animals of the same type were compared at the ages of 20 and 26 weeks to assess aging effects in the control lean ZDF (+/+) rats and disease progression in the obese diabetic ZDF (fa/fa) ones. Comparisons were made between lean (+/+) and obese diabetic (fa/fa) ZDF rats, at 20 and 26 weeks, to differentiate diabetic features from normal (20 weeks) or ageing characteristics (26 weeks). After these prior comparisons, our main group of interest, the chronic sitagliptin-treated obese diabetic (fa/fa) ZDF rats of 26 weeks, were compared to its untreated counterparts. Food intake and BW were measured each day before treatment and expressed as weekly average values.

### 2.2. Sample Collection and Preparation

**Blood**

When aged 20 weeks (T0) and at the end of the experiment (26 weeks-Tf), the rats were subjected to intraperitoneal anesthesia with a 2 mg/kg BW of a 2 : 1 (v : v) 50 mg/mL ketamine (Ketalar, Parke-Davis, Lab. Pfeizer Lda, Seixal, Portugal) solution in 2.5% chlorpromazine (Largactil, Rhône-Poulenc Rorer, Lab. Vitória, Amadora, Portugal), and blood samples were immediately collected by venipuncture from the jugular vein into syringes without anticoagulant (for serum samples) or with the appropriate anticoagulant: ethylene-diaminetetraacetic acid (EDTA)-2K for Glycosylated haemoglobin (HbA_1_c) measurement.

**Tissues**

The rats were sacrificed by anesthetic overdose. The kidneys were immediately removed, placed in ice-cold Krebs' buffer and carefully cleaned of extraneous fat and connective tissue. Subsequently, the organ was cross-sectioned, fixed and processed for paraffin embedding in accordance with the subsequent histological protocols.

### 2.3. Glycaemic, Insulinaemic and Lipidic Profile Assays

Serum glucose levels were measured using a Glucose oxidase commercial kit (Sigma, St. Louis, Mo, USA). Considering the variability of serum glucose levels in the rat, glycosylated haemoglobin (HbA_1_c) levels were used as an index of glucose control, through the DCA 2000+ latex immunoagglutination method (Bayer Diagnostics, Barcelona, Spain). Plasma insulin levels were quantified by using a rat insulin ELISA assay kit from Mercodia (Uppsala, Sweden). The steady state beta cell function of individual animals was evaluated using the previously validated homeostasis model assessment (HOMA) of *β*-cell function [35]. The formula used was as follow: [HOMA-*β*%] = 360 × fasting serum insulin (mU/L)/fasting serum glucose (mg/dL)−63. The values used (insulin and glucose) were obtained after an overnight of food deprivation. Serum total cholesterol (Total-c) and triglycerides (TGs) were analysed on a Hitachi 717 analyser (Roche Diagnostics) using standard laboratorial methods. Total-c reagents and TGs kit were obtained from bioMérieux (Lyon, France).

### 2.4. Kidney Function (Creatinine and Urea) and Trophism (Weight)

Serum creatinine and blood urea nitrogen (BUN) concentrations were used as renal function indexes, through automatic validated methods and equipments (Hitachi 717 analyser, Roche Diagnostics Inc., Mass, USA). The weights of kidneys (KW) and the ratio KW/BW were measured in all the rats under study in order to be used as renal trophy indexes.

### 2.5. Kidney Lipid Peroxidation

Kidney lipid peroxidation was assessed by the thiobarbituric acid reactive-species (TBARs) assay, measuring the malondialdehyde (MDA) content, according to that previously described in [32]. Samples were analysed spectrophotometrically at 532 nm using 1,1,3,3-tetramethoxypropane as external standard. The concentration of lipid peroxides (in MDA) was expressed as *μ*mol/L.

### 2.6. Histopathological Analysis

**Haematoxylin and Eosin Staining**

Samples were fixed in Bock's fixative and embedded in paraffin wax, and 3 *μ*m thick sections were stained for routine histopathological diagnosis with haematoxylin and eosin (HE).

**Periodic Acid of Schiff Staining**

Periodic acid of Shiff (PAS) was used to evaluate and confirm the levels of mesangial expansion, thickening of basement membranes and sclerotic parameters. Samples were fixed in neutral formalin 10%, embedded in paraffin wax, and 3 *μ*m thick sections were immersed in water and subsequently treated with a 1% aqueous solution of periodic acid, then washed to remove any traces of the periodic acid, and finally treated with Schiff's reagent. All samples were examined by light microscopy using a Microscope Zeiss Mod. Axioplan 2. The degree of injury visible by light microscopy was scored in a double-blinded fashion by two pathologists. Lesions were evaluated on the total tissue on the slide.

**Histopathology**

Glomerular damage was assessed by evaluating mesangial expansion, glomerular basement membrane and capsule of Bowman thickening, nodular sclerosis, glomerulosclerosis, atrophy, and hyalinosis of the vascular pole. Analysed tubulointerstitial lesions comprised inflammation, presence of hyaline cylinders, tubular basement membrane irregularity, tubular calcification, and the association of interstitial fibrosis and tubular atrophy (IFTA). The evaluation of vascular lesions was concentrated on arteriolar hyalinosis and arteriosclerosis. A semiquantitative rating for each slide ranging from normal (or minimal) to severe (extensive damage) was assigned to each component. Severity was graded as absent/normal, mild, moderate, and severe. Scoring was defined according to the extension occupied by the lesion (% area): normal: <25%; mild: 25–50%; moderate: 50–75%; severe: >75%. The final score of each sample was obtained by the average of scores observed in individual glomeruli in the considered microscopic fields. Tubulointerstitial damage was evaluated and graded by the same semiquantitative method, with the exception of IFTA, which was graded as normal, if absent, as mild, moderate, and severe, if present in <25%, between 25–50%, and over 50% of affected area. Regarding vascular lesions, arteriolar hyalinosis was scored as 0 if absent, as 1 if one arteriole with hyalinosis was present, and as 2 if more than one arteriole was observed in the entire slide. Arteriosclerosis was scored as 0 if no intimal thickening was present, as 1 if intimal thickening was less than the thickness of the media, and as 2 if intimal thickening was more than the thickness of the media and considering the worst artery on the slide. When using PAS, the rating was set for intensity and extension of staining, ranging from 0 (no staining) to 3 (intense and extensive staining), respecting tissue specificity scoring when adequate.

### 2.7. Statistical Analysis

The categorical variables are counts of renal lesions severity in scores. Quantitative values are reported as mean ± SEM. Significance level was accepted at 0.05. Data were analyzed using SPSS Statistics 18 (2009). Chi-square test with Monte Carlo simulation or exact test (when contingency tables are 2 × 2) was used to find out the differences of severity score distributions in renal lesions at the beginning of the study (20 weeks old) between lean control and obese diabetic ZDF (fa/fa) rats and at the end of the study (26 weeks old), between diabetic ZDF (fa/fa) rats vehicle-treated and diabetic ZDF (fa/fa) sitagliptin-treated and lean control rats. To get an overview of the influence of sitagliptin treatment in renal lesions after 6 weeks of chronic treatment with sitagliptin (final time 26 weeks), we generated two quantitative variables, by averaging the scores of two types of renal lesions: global glomerular lesions comprising mesangial expansion, thickening of GBM, thickening of CB, nodular sclerosis, glomerulosclerosis, glomerular atrophy, and hyalinosis of the vascular pole and global tubulointerstitial lesions comprising hyaline cylinders, TBM irregularity, tubular calcification, IFTA, and tubular degeneration. On these two variables was performed an ANOVA and subsequent LSD post hoc test to find out the differences between diabetic ZDF (fa/fa) rats vehicle treated, diabetic ZDF (fa/fa) rats sitagliptin treated, and lean control rats.

## 3. Results

### 3.1. Effects of Sitagliptin Treatment on Body Weight and Glycaemic and Lipidic Profiles

Concerning the body weight, no significant differences were encountered between the diabetic and the lean control rats in the beginning of treatments (T0: week 20) despite the obese profile encountered in the diabetic ZDF (fa/fa) rats between the 8th and the 14th week (data not shown). At the end of the study (26 weeks), the control diabetic ZDF (fa/fa) rats exhibit an 8.7% reduction in their BW (*P* < 0.001); nevertheless, the lean control group gained weight. Sitagliptin treatment, during 6 weeks, stabilized the loss of weight in the diabetic ZDF (fa/fa) rats even preventing part of the BW loss when compared with the rats without treatment (Table 1). 

At the T0 (when animals aged 20 weeks), the diabetic group showed a hyperglycaemic and a hyperlipidemic profile, also seen at the final time (Table 1). The values of HbA1c were higher in the diabetic rats when compared with those of the control animals, confirming the glycaemic deregulation. The diabetic ZDF (fa/fa) rats have also presented higher levels of Total-c and TGs versus the control ZDF (+/+) animals, in both times (Table 1). After 6 weeks of sitagliptin treatment (Tf: 26 weeks), a significant (*P* < 0.001) improvement in glycaemic control was observed in diabetic ZDF (fa/fa) rats, when compared with the vehicle-treated counterparts. This pattern of changes is also expressed by the HbA1c levels. TGs were significantly reduced (*P* < 0.001) in the diabetic rats treated with sitagliptin during 6 weeks versus the diabetic vehicle-treated animals (Table 1).

### 3.2. Effects of Sitagliptin Treatment on Insulin Levels and HOMA-Beta

At the beginning of the study (Ti: 20 weeks age), insulin levels were already significantly lower in diabetic animals when compared to lean control (*P* < 0.01) together with a significant different value of HOMA-beta (*P* < 0.001) (Table 1). At the final time, the vehicle-treated diabetic ZDF (fa/fa) rats exhibit relative insulinopaenia, when compared to vehicle-treated ZDF (+/+), accompanied by a further decrease (*P* < 0.001) of HOMA-beta. The insulinopaenic profile of the diabetic rats, as well as the decrease of HOMA-beta value, were partially, significantly prevented (*P* < 0.001) in the diabetic rats treated with sitagliptin when compared with those untreated (Table 1).

### 3.3. Effects of Sitagliptin Treatment on Kidney Function (Creatinine and Urea) and Trophism (Weight)

At the beginning of the study (T0), urea contents were already significantly higher (*P* < 0.001) in the diabetic ZDF rats when compared with the control animals, without significant changes of creatinine (Table 2). The diabetic rats treated with sitagliptin showed urea values identical to those found for the control animals at the final time (26 weeks), contrasting with the higher value (*P* < 0.01) encountered in the diabetic ZDF without treatment (Table 2). Concerning the kidney trophism, we found that at week 20 (T0), there was already kidney hypertrophism, viewed by increased value (*P* < 0.05) of KW and of KW/BW in the diabetic rats when compared with the control animals, which was even increased in the final time (Table 2). Sitagliptin treatment did not changed kidney trophism parameters in the diabetic animals (Table 2).

### 3.4. Effects of Sitagliptin Treatment on Kidney Lipidic Peroxidation

**Figure 1:**
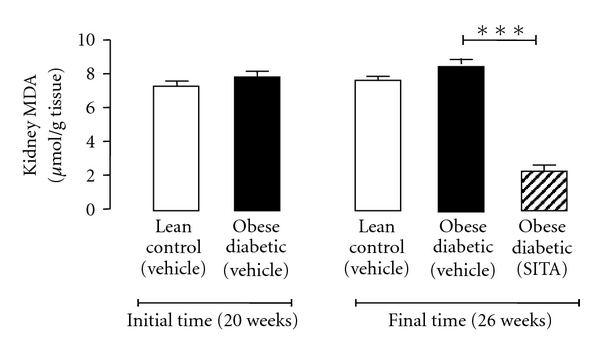
Kidney lipidic peroxidation (MDA) for the lean control and obese diabetic ZDF rats, in the initial and final times (6 weeks of vehicle or 10 mg/kg BW/day sitagliptin treatment). Data is expressed as mean ± sem of 8 rats/group: ***P < 0.001. MDA, malondialdehyde; SITA, sitagliptin.

At the initial time (20 weeks), MDA contents were unchanged between the lean control and the diabetic animals. A trend to higher values in the diabetic rats was found at the final time (Tf). This profile was completely reversed by sitagliptin treatment, since the kidney MDA values were substantially (*P* < 0.001) lower than those found in the diabetic untreated animals (Figure 1).

### 3.5. Effects of Sitagliptin Treatment on Renal Lesions Evolution

#### 3.5.1. Glomerular Lesions

**Figure 2:**
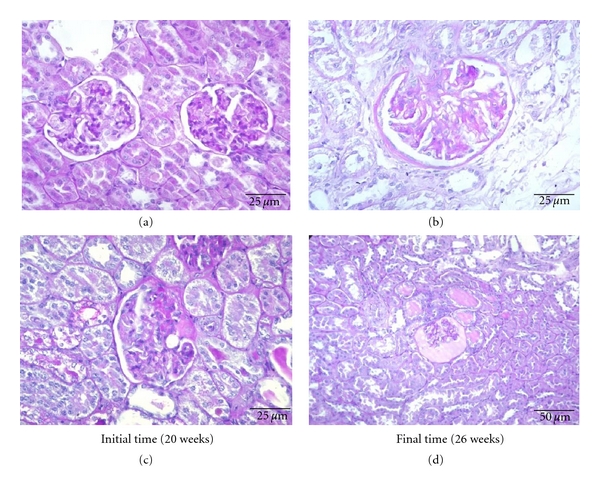
Evolution of renal lesions with ageing in lean control and obese diabetic ZDF rats: (a) normal renal histology in a lean control rat at 20 weeks of age (PAS, 400x); (b) a glomerulus presenting grade 1 mesangial expansion and thickening of the capsule of Bowman in a lean control rat at 26 weeks of age (PAS, 400x); (c) nodular glomerulosclerosis with sinequia of the tuft to Bowman's capsule, mesangial expansion and arteriolar sclerosis in a diabetic rat of 20 weeks (PAS, 400x); (d) atrophic, sclerosed glomerulus, exhibiting filtrate fluid in Bowman's space. Note the presence of hyaline cylinders and the irregularity of tubular basement membranes, diabetic rat of 26 weeks (PAS, 200x).

Comparative analysis between lean control and obese diabetic ZDF rats of 20 weeks of age revealed a significantly (*P* < 0.001) increased mesangial expansion, nodular sclerosis, glomerulosclerosis, and glomerular atrophy in the obese diabetic animals, accompanied by a significant thickening of glomerular basement membrane and capsule of Bowman (*P* < 0.01) (Figures 2(a) and 2(c)). When aged 26 weeks, the obese diabetic rats showed aggravated glomerular basement membrane thickening and glomerular atrophy (*P* < 0.001), when compared with the lean control animals, accompanied by a significantly more intense expression of mesangial expansion and capsule of Bowman thickening (*P* < 0.01). Glomerulosclerosis was also significantly more obvious in diabetic subjects (*P* < 0.05) (Figures 2(b) and 2(d)). Concerning ageing effects from 20 to 26 weeks in the lean rats, the most noted alterations were capsule of Bowman thickening (*P* < 0.01) and increase in nodular sclerosis (*P* < 0.01) (Figures 2(a) and 2(b)). In the obese diabetic rats, between 20 and 26 weeks, there was a statistically significant increase in glomerular basement membrane (*P* < 0.05) and capsule of Bowman thickening (*P* < 0.05). Hyalinosis of the vascular glomerular pole was absent in all lean rats but was present in the obese diabetic ZDF rats, as soon as 20 weeks of age, with a tendency for aggravation in the 26 weeks (data not shown).

**Figure 3:**
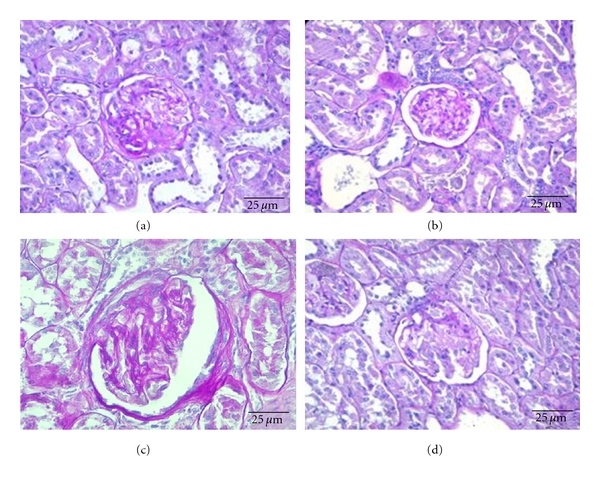
Effects of chronic sitagliptin treatment on renal lesions in obese diabetic ZDF rats; (a) regression of glomerulosclerosis, with more glomeruli presenting the more benign nodular form of sclerosis; (b) reduction in capsule of Bowman thickness and absence of sclerosis; (c) although there is persistence of grade 2 capsular thickening, there is absence of sclerosis and only the presence of grade 1 mesangial expansion; (d) presence of light mesangial expansion and hyalinosis of the vascular pole. Note in all figures the absence of hyaline cylinders and a more regular contour of the tubular basement membranes PAS, 400x.

**Figure 4:**
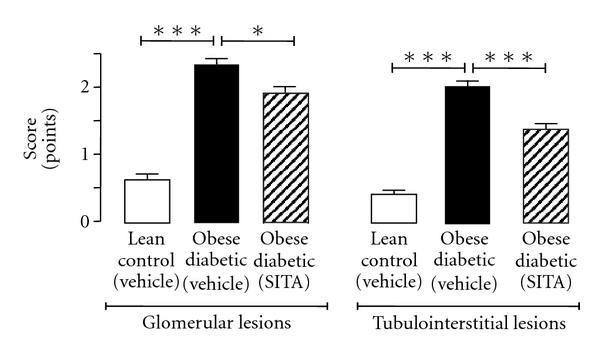
Effects of chronic sitagliptin treatment on renal glomerular and tubulointerstitial lesions in obese diabetic ZDF rats, at the final time (26 weeks). Data is expressed as mean ± sem of 8 rats/group: *P < 0.05 and ***P < 0.001. SITA, sitagliptin.

Concerning the sitagliptin effects in the diabetic rats at 26 weeks old, there was a reduction of severity of fibrosis, demonstrated by the significant decrease of global glomerulosclerosis (*P* < 0.01), which is in agreement with the less severe nodular sclerosis (*P* < 0.01) (Figure 3(a)). Hyalinosis of the vascular glomerular pole was also significantly decreased (Table 3). Mesangial expansion, glomerular atrophy, and glomerular basement membrane thickening showed a trend to improvement in the sitagliptin-treated diabetic rats versus the untreated (Figures 3(b), 3(c), and 3(d) and Table 3). Therefore, mesangial expansion showed a 37.5% reduction in the most severe grade; glomerular atrophy and glomerular basement membrane presented a 25% and 12.5% reduction, respectively, in grade 2 and 3 of lesion severity (Table 3). When considering all the glomerular lesions, the diabetic rats presented a notorious pattern of lesion (*P* < 0.001), when compared with the lean animals, which was significantly ameliorated (*P* < 0.05) by chronic sitagliptin treatment (Figure 4).

#### 3.5.2. Tubulointerstitial Lesions

When aged 20 weeks, the obese diabetic rats already presented a significant increase in tubular degeneration (*P* < 0.01), tubular basement membrane irregularity, and IFTA (*P* < 0.01), when compared with the lean controls animals. The differences between these groups were more pronounced when aged 26 weeks, in which the obese diabetic subjects showed marked aggravation of hyaline cylinders, tubular basement membrane irregularity and IFTA (*P* < 0.001), together with significant increase in tubular degeneration (*P* < 0.01) (Table 4). The most significant ageing alterations found in the lean rats were tubular basement membrane irregularity (*P* < 0.01) and IFTA (*P* < 0.01), while in the obese diabetic animals, these were mainly IFTA (*P* < 0.001) and hyaline cylinders (*P* < 0.01) aggravation (Figure 2(d)).

Sitagliptin significantly prevented the appearance of hyaline cylinders in chronically treated diabetic rats (*P* < 0.001), together with a trend to decreased basement membrane irregularity (by 50%), tubular degeneration, and IFTA (by 37.5%) in grade 3 of lesion severity (Figure 3 and Table 4). Calcification of tubular epithelium was only present in diabetic rats, which did not suffer any mentionable recovery with sitagliptin treatment (Table 4). When considering all the tubulointerstetial lesions, the diabetic rats presented a pattern of lesion (*P* < 0.001), when compared with the lean animals, which was significantly ameliorated (*P* < 0.001) by chronic sitagliptin treatment (Figure 4).

#### 3.5.3. Vascular Lesions

Arteriolar hyalinosis was only found in the diabetic rats, which aggravated between 20 and 26 weeks (*P* < 0.05). Arteriosclerosis was only detected in lean animals when aged 26 weeks but was present in the diabetic rats at 20 weeks, which also exhibited aggravation of sclerosis at the final time, with 62.5% of the animals exhibiting grade 1 and 25% grade 2 lesions, in comparison to its lean counterparts, which showed 50% of animals in grade 1 and none in grade 2 (Table 5). Sitagliptin promoted a 50% improvement in the most severe form of hyalinosis (grade 2) and reduced the incidence of arteriosclerosis in the treated diabetic rats by 12.5% (Table 5).

## 4. Discussion

Diabetic nephropathy has emerged as the leading cause of end-stage renal disease (ESRD), and thus, preventing or delaying it, has been a major goal in biomedical research. The development of innovative therapeutic alternatives, such as the incretin enhancers (including sitagliptin), able to target not only hyperglycaemia, but also multiple risk factors, seems more likely to be beneficial as shown by recent approaches [27, 32]. Our present study reports the progression of renal disease in ZDF rats and demonstrates that a daily chronic administration of low-dose sitagliptin markedly reduces renal injury in this model.

It is well known that a commonly accepted animal model for type 2 diabetic nephropathy has not been available. The ZDF rat is characterized by hyperglycaemia, hyperinsulinaemia, hyperlipidaemia, moderate hypertension and obesity, and progressive renal injury [29]. These rats develop nephropathy by 12 wks of age, earlier than in most of other models of type 2 diabetes, characterized by focal segmental glomerulosclerosis (FSGS), associated with glomerulomegaly and mesangial expansion [36]. Thus, this animal model seems to be useful for preclinical evaluation of novel pharmacological compounds in human diabetic nephropathy. In the present study, the animal's ages were selected according to moment of initiation of relative insulinopenia (20 weeks) and of presence of significant diabetic complications (26 weeks). Although the literature describes in this animal model an earlier nephropathy, our animals were fed with normal rodent maintenance chow (with 2.9% of lipids) for developing all the different stages of T2DM in latter times than those described for this animal model. Therefore, if we intend to analyse renal lesions when rats presented lower insulin levels, those are the proper animal's ages. In order to achieve a better correlation between our animal observations and the human nephropathy process, we decided to adapt a recent human pathologic classification for diabetic nephropathy [37]. Despite the fact that our untreated diabetic ZDF presented lower body weight (BW) than their lean counterparts, our data show that along with the metabolic changes occurring over time in these rats, the nephropathy resembles human diabetic nephropathy in terms of morphology. The significant body weight loss of ZDF diabetic rats corresponds to the time of significant depletion of serum insulin levels compared with age-matched lean ZDF rats, which was an expected profile and is in agreement with the aggravation of the disease.

We must empathize that the administration of 10 mg/kg/day of sitagliptin, used in the current study, may be considered a low dose, as others have used higher doses or the administration of 10 mg/kg/BW twice a day [38, 39]. Nevertheless, we took in consideration that renal toxicity is very likely related to the extremely high urinary concentrations that result from rapid renal elimination of the drug in rodents. Since sitagliptin is virtually completely absorbed following an oral dose in rodents [40], the initial body burden of the drug is likely to be more directly related to the dosage on a mg/kg body weight basis than on a plasma AUC.

The nephropathy in this model has previously been described as focal segmental glomerulosclerosis (FSGS) associated with glomerulomegaly and mesangial expansion, findings characteristically seen in patients with obesity and metabolic syndrome [41, 42] associated with T2DM. In the literature, we found the descriptions of the tubulointerstitial lesions are mentioned only in passing and as secondary pathology [36, 43]. Renal vascular pathology has not been described. The data presented herein provides morphologic characterization of progressive nephropathy, including the glomerular, tubulointerstitial, and vascular lesions in the kidney of ZDF rats. 

The lean ZDF rats demonstrated at 20 wk thickening of GBM, mesangial expansion, nodular sclerosis, interstitial fibrosis, and tubular atrophy (IFTA), which further aggravates with age. These observations are in accordance with Vora et al. (1996) [44] and could be classified as nondiabetic renal lesions attributed to aging in this strain. All the obese diabetic ZDF rats presented significant glomerular, tubulointerstitial and vascular lesions compared with lean ZDF controls in both ages analysed (20 and 26 wks). In the obese diabetic ZDF rats, the severity of the lesions aggravates with diabetes progression, confirming a link between diabetes (hyperglicaemia and hyperlipidaemia) and progressive renal injury. 

In patients with diabetic nephropathy, the initial physiological change is glomerular hyperfiltration, while the initial morphological change is glomerular hypertrophy. At 26 wks old, the obese ZDF rats exhibit an aggravation of the lesions described for 20 wks, including mesangial expansion, glomerular basement membrane thickening, and glomerular hypertrophy. We observed that tubulointerstitial lesions are dependent of glomerulosclerosis, which is suggested by the aggravation of both (glomeruli and interstitium). Vascular pole hyalinization and arthrosclerosis also suffer aggravation with age. All of these histological alterations were accompanied by an augmentation of kidney weight. In the obese diabetic ZDF rats, a glomerular hypertrophy, expansion in the mesangial area related to the mesangial matrix, and renal hypertrophy was noted. In the present study, we did not evaluate the progression of proteinuria, but it is well documented by others [45, 46]. We measured blood urea nitrogen (BUN), and the results showed a significant increase the obese diabetic ZDF rats when compared to the lean control, suggesting a deficient kidney function. Nevertheless, serum creatinine levels were unchanged between groups, which is in accordance with others [47]. 

Chronic sitagliptin (low-dose) treatment ameliorated all lesions (glomerular, tubulointerstitial, and vascular), except the tubular epithelium calcification, in the diabetic-treated rats. Chronic sitagliptin administration was able to decrease BNU to levels analogous to those observed in lean controls, suggesting an amelioration of kidney function. The mechanism by which a low-dose of sitagliptin, which was unable to completely normalize the hyperglycaemic profile of the diabetic rats, is able to positively modulate kidney function is unknown. We may hypothesize that significant improvement of circulating levels of TG result in the attenuation of renal injury in treated diabetic ZDF rats. One explanation for this is that the augment of insulin levels by sitagliptin inhibits adipose tissue hormone-sensitive lipase (HSL) activity and, thus, adipose tissue fatty acid release. In addition, insulin and the augment of GIP induced by DPP-4 inhibition may enhance adipose tissue fatty acid reesterification and, thus, increase adipose tissue triacylglycerol (TAG) deposition. In the present work, we did not measure fat pads in ZDF rats, we did not evaluated lipids in kidney, and, thus, we cannot confirm our hypothesis. Nevertheless, in future studies, we intend to perform oil red staining in the kidney in order to assess lipotoxicity and the putative effects of sitagliptin. However, some previous data from our studies should be mentioned. We have demonstrated that this low-dose chronic sitagliptin treatment is able to promote a favorable impact on chronic inflammation and oxidative stress, which are key players of diabetes pathophysiology and may precede and further potentiate tissue damage [32]. Despite the lower dose used, we have previously demonstrated beneficial effects of sitagliptin on metabolic profile and reduction in inflammatory markers, as well as an amelioration of fibrosis, vacuolization, and congestion in endocrine pancreas and preservation of pancreatic islets were previously suggested [32]. The histomorphological observations were in accordance with the improvement in pancreatic beta-cell function, as suggested by the sitagliptin-evoked augment in HOMA-beta. The effects of chronic DPP-4 inhibition in increasing *β*-cell mass and function over time may occur, at least in part, by the augmentation of glucose-stimulated insulin secretion. This effect is believed to be primarily mediated via stabilization of the incretin hormones contents, including of GLP-1 [48]. We also observed a weight gain of treated diabetic animals that could be attributed to the amelioration induced by sitagliptin in the dysmetabolism and thus to an improvement in general condition. This metabolic improvement by sitagliptin in diabetic ZDF rats was accompanied by a reduction in inflammatory markers (CRP and IL-1 beta) and pancreatic oxidative stress, as previously documented by our group [32]. Our results agree with those performed by others, which have been suggesting an antioxidant and anti-inflammatory effect of incretin modulators, due to attenuation of the deleterious effects of AGEs-RAGE-oxidative stress axis and to protection against the cytokine-induced apoptosis and necrosis [49–51]. 

Although large body of evidence indicates that oxidative stress is involved in the progression of fibrosis and end-stage renal disease, in experimental and human diabetic nephropathy [52], we failed to demonstrate it, at least when comparing kidney lipid peroxidation between diabetic untreated ZDF rats their lean mach control. However, further studies should better address this aspect, namely, by assessing other relevant kidney markers of oxidative stress, including levels of AGEs, as well as contents of antioxidants. However, our work suggests a favourable impact of sitagliptin treatment on kidney oxidative stress profile, expressed by reduced amount of lipid peroxidation, which might be further confirmed with additional parameters, but that is in agreement with recent studies from Vaghasiya et al. (2011) which have reported a significant decrease in renal lipidic peroxidation by sitagliptin in diabetic rats with renal damage [53]. 

Experimental evidence linking hyperlipidaemia to renal injury and progression of renal fibrogenesis has been well documented; lipids can modulate the progression of chronic renal diseases and may even be primary factors in the pathogenesis of renal tissue injury [54]. Additionally, the synergistic effects of hyperlipidaemia and diabetes on the development of renal injury have been recently observed in several animal models [55, 56]. In ZDF rats, Chander et al. (2004) and Suzaki et al. (2006) suggested that hyperlipidaemia, in concert with hyperglycaemia, may be responsible for the increased oxidative stress and initiation and aggravation of injury in the kidneys of these animals [57, 58]. Thus, we may hypothesize that the ability of sitagliptin to lower plasma lipids, as well as to promote a more favorable redox status in the kidney, as confirmed in the present study by the reduction of lipid peroxidation products, may have contributed to its renoprotective effects. Furthermore, the positive effects demonstrated in peripheral insulin resistance and pancreas lesions, as well as the antihypertensive effect [32], might be viewed as probable contributors to the renoprotection described in this study. On the other hand, we could not exclude the possible effects of the expected sitagliptin-induced inhibition of DPP-4 and consequent increment of GLP-1, since these effects have been associated by others to a protection of mesangial cells and to an amelioration of sodium, acid-base, and fluid homeostasis that contributes to the renoprotection [59, 60]. In any case, future studies should confirm the effects of sitagliptin on DPP-4 activity/expression, as well as on GLP-1 and glucagon levels, in order to have a more detailed picture of how the incretin pathway is affected and its relative contribution for the effects of sitagliptin here reported.

To our knowledge, this is the first report on the amelioration of diabetic nephropathy, and specifically of glomerulosclerosis, tubulointerstitial and vascular kidney lesions, by a chronic administration of a low dose of sitagliptin that does not reduce hyperglycaemia below a rather high level (partial, but significant, correction), indicative of noncompensated diabetes. The present study demonstrated that sitagliptin delays the development of nephropathy in ZDF rats, concomitantly with hypoglicaemic, hypolipidaemic and antioxidant effects. Although, further studies are required to elucidate the nature of the protective effects of sitagliptin on the diabetic kidney, the obtained results are consistent with pleiotropic effects of this new antidiabetic drug, which might underlie the renoprotective properties.

## 5. Conclusions

Chronic administration of a low dose of sitagliptin was able to ameliorate diabetic nephropathy in this model of obese type 2 diabetes/nephropathy, viewed by significant reduction of glomerulosclerosis and tubulointerstitial and vascular kidney lesions, which might be partial due to its benefits on correction of diabetes dysmetabolism (hyperglicaemia, dyslipidaemia, and insulin production/sensitivity), and due to a favorable impact on kidney lipid peroxidation. Further studies are required to assess the cellular/molecular nature of these effects. However, the beneficial and novel profile of this incretin modulator could prove crucial in the prevention of diabetic nephropathy evolution and might represent a key step forward in the management of T2DM and this serious complication.

## Figures and Tables

**Table 1:** Body weight, glycaemic, insulinaemic and lipidic profile in the lean control and diabetic ZDF rats at the initial and final time (6 weeks of vehicle or sitagliptin treatment).

Time	Initial time (20 wks)		Final time (26 wks)		
Rat group	Lean control	Obese diabetic	Lean control	Obese diabetic	
Parameters	(*n* = 16)	(*n* = 16)	Vehicle (*n* = 8)	Vehicle (*n* = 8)	SITA (*n* = 8)
BW (g)	406.70 ± 6.83	388.10 ± 8.87	445.70 ± 8.16	354.40 ± 8.85^aaa^	380.00 ± 14.46
Glucose (mg/dL)	133.30 ± 1.20	523.30 ± 3.60^aaa^	133.30 ± 1.20	633.1 ± 15.70^aaa^	546.33 ± 19.30^bbb^
HbA1c (%)	3.16 ± 0.12	10.38 ± 0.50^aaa^	3.20 ± 0.14	10.96 ± 0.20^aaa^	9.18 ± 0.75^bbb^
Insulin (mU/L)	15.00 ± 5.90	13.70 ± 0.90^aa^	15.80 ± 3.00	7.60 ± 1.50^aaa^	10.60 ± 1.80^bbb^
HOMA-Beta (%)	76.80 ± 4.05	13.84 ± 1.50^aaa^	80.90 ± 7.56	4.80 ± 1.12^aaa^	7.89 ± 0.97^bbb^
Total-c (mg/dL)	77.50 ± 1.50	155.50 ± 3.50^aaa^	93.00 ± 2.96	193.00 ± 9.79^aaa^	193.10 ± 4.62
TGs (mg/dL)	115.00 ± 11.00	374.50 ± 4.95^a^	154.00 ± 19.14	400.20 ± 27.00^aaa^	237.10 ± 22.54^bbb^

Values are means ± SEM of *n* rats. ^a^Lean control (vehicle) versus obese diabetic (vehicle) rats; ^b^diabetic SITA-treated versus diabetic untreated rats. One, two, or three letters for *P* < 0.05, *P* < 0.01, and *P* < 0.001, respectively. BW: body weight; HbA1c: glycosylated haemoglobin; HOMA: homeostasis model assessment; SITA: sitagliptin; Total-c: total-cholesterol; TGs: triglycerides; ZDF: Zucker diabetic fatty.

**Table 2:** Assessment of kidney function (serum creatinine and BUN) and weights (trophism) in the lean control and diabetic ZDF rats at the initial and final time (6 weeks of vehicle or sitagliptin treatment).

Time	Initial time (20 wks)		Final time (26 wks)		
Rat group	Lean control	Obese diabetic	Lean control	Obese diabetic	
Parameters	(*n* = 16)	(*n* = 16)	Vehicle (*n* = 8)	Vehicle (*n* = 8)	SITA (*n* = 8)
Creatinine (mg/dL)	0.55 ± 0.03	0.55 ± 0.06	0.53 ± 0.03	0.54 ± 0.08	0.49 ± 0.04
BUN (*μ*g/L)	14.35 ± 0.47	18.15 ± 0.84^aaa^	15.05 ± 0.54	18.03 ± 1.20^aa^	15.16 ± 0.61^b^
KW (g)	2.39 ± 0.08	3.25 ± 0.26^a^	2.56 ± 0.04	3.02 ± 0.09^a^	3.15 ± 0.05
KW/BW (g/Kg)	6.11 ± 0.15	8.82 ± 0.73^a^	5.71 ± 0.07	8.42 ± 0.42^aaa^	8.42 ± 0.40

Values are means ± SEM of *n* rats. Comparisons between groups: ^a^lean control (vehicle) versus obese diabetic (vehicle) rats; ^b^diabetic SITA-treated versus diabetic untreated rats. One, two, or three letters for *P* < 0.05, *P* < 0.01, and *P* < 0.001, respectively. BUN: blood urea nitrogen; BW: body weight; KW: kidney weight; SITA: sitagliptin; ZDF: Zucker diabetic fatty.

**Table 3:** Scoring and distribution of glomerular lesions in lean control and obese diabetic ZDF rats kidneys at the final time, 26 weeks of age (6 weeks of vehicle or sitagliptin treatment).

lomerular lesion	Rat group	Scoring and distribution of glomerular lesions (*n* of rats)			
	(*n* = 8 each)	Normal	Mild	Moderate	Severe
Mesangial expansion	Lean control (vehicle)	3	3	2	0
	Obese diabetic (vehicle)	0	0	3	5^aaa^
	Obese diabetic (SITA)	0	1	5	2

Thickening of GBM	Lean control (vehicle)	3	5	0	0
	Obese diabetic (vehicle)	0	0	2^aa^	6^aa^
	Obese diabetic (SITA)	0	2	1	5

Thickening of CB	Lean control (vehicle)	1	6	1	0
	Obese diabetic (vehicle)	0	0	4	4
	Obese diabetic (SITA)	0	4	0	4

Nodular sclerosis	Lean control (vehicle)	2	4	2	0
	Obese diabetic (vehicle)	0	5	3	0
	Obese diabetic (SITA)	0	0	2^bb^	6^bb^

Glomerulosclerosis	Lean control (vehicle)	2	3	3	0
	Obese diabetic (vehicle)	0	0^a^	3	5^aaa^
	Obese diabetic (SITA)	0	4^bb^	4^b^	0^bbb^

Glomerular atrophy	Lean control (vehicle)	6	2	0	0
	Obese diabetic (vehicle)	0	0	4^aaa^	4^aaa^
	Obese diabetic (SITA)	0	4^bb^	2^b^	2^b^

Hyalinosis of the vascular pole	Lean control (vehicle)	8	0	0	0
	Obese diabetic (vehicle)	2	1	2	3
	Obese diabetic (SITA)	0	7^bbb^	1^b^	0^b^

^a^Lean control (vehicle) versus obese diabetic (vehicle) rats; ^b^diabetic SITA-treated versus diabetic untreated rats. One, two, or three letters for *P* < 0.05, *P* < 0.01, and *P* < 0.001, respectively. CB: capsule of Bowman; GMB: glomerular basement membrane; SITA: sitagliptin. Scoring was defined according to the extension occupied by the lesion (% area of the glomerulus): normal: <25%; mild: 25–50%; moderate: 50–75%; severe: >75%.

**Table 4:** Scoring and distribution of tubular lesions in lean control and obese diabetic ZDF rats kidneys at the final time, 26 weeks of age (6 weeks of vehicle or sitagliptin treatment).

Tubular lesion	Rat group	Scoring and distribution of tubular lesions (*n* of rats)			
	(*n* = 8 each)	Normal	Mild	Moderate	Severe
Hyaline cylinders	Lean control (vehicle)	6	2	0	0
	Obese diabetic (vehicle)	0	0	7^aaa^	1
	Obese diabetic (SITA)	0	8^bb^	0	0

TBM irregularity	Lean control (vehicle)	2	5	1	0
	Obese diabetic (vehicle)	0	0	1	7^aaa^
	Obese diabetic (SITA)	0	3^bb^	2	3^bb^

Tubular calcification	Lean control (vehicle)	8	0	0	0
	Obese diabetic (vehicle)	5	3	0	0
	Obese diabetic (SITA)	4	4	0	0

IFTA	Lean control (vehicle)	2	6	0	0
	Obese diabetic (vehicle)	0	0	3^aa^	5^aaa^
	Obese diabetic (SITA)	1	2	3	2^bb^

Tubular degeneration	Lean control (vehicle)	4	4	0	0
	Obese diabetic (vehicle)	0	1^aa^	4^aaa^	3^aaa^
	Obese diabetic (SITA)	0	3^b^	5^b^	0^bb^

^a^Lean control (vehicle) versus obese diabetic (vehicle) rats; ^b^diabetic SITA-treated versus diabetic untreated rats. One, two, or three letters for *P* < 0.05, *P* < 0.01, and *P* < 0.001, respectively. TMB: tubular basement membrane; IFTA: interstitial fibrosis and tubular atrophy. Scoring was defined according to the extension occupied by the lesion (% area of the tubulus): normal: <25%; mild: 25–50%; moderate: 50–75%; severe: >75%. SITA, sitagliptin.

**Table 5:** Scoring and distribution of vascular lesions in lean control and obese diabetic ZDF rats kidneys at the final time (26 weeks of age). Diabetic ZDF rats with versus without chronic sitagliptin.

Vascular lesion	Rat group	Scoring and distribution of vascular lesions (*n* of rats)		
	(*n* = 8 each)	Normal	Mild/moderate	Severe
Arteriolar hyalinosis	Lean control (vehicle)	8	0	0
	Obese diabetic (vehicle)	1^aa^	1	6^aa^
	Obese diabetic (SITA)	3	3^b^	2^b^

Arteriosclerosis	Lean control (vehicle)	4	4	0
	Obese diabetic (vehicle)	1	5	2
	Obese diabetic (SITA)	3	4	1

^a^Lean control (vehicle) versus obese diabetic (vehicle) rats; ^b^diabetic SITA-treated versus diabetic untreated rats. One, two or three letters for *P* < 0.05, *P* < 0.01, and *P* < 0.001, respectively. Scoring was defined according to the following criteria: arteriolar hyalinosis was scored as 0 if absent, as 1 if one arteriole with hyalinosis was present, and as 2 if more than one arteriole was observed in the entire slide. Arteriosclerosis was scored as 0 if no intimal thickening was present, as 1 if intimal thickening was less than the thickness of the media, and as 2 if intimal thickening was more than the thickness of the media. SITA, sitagliptin.
